# Humpback whale song complexity and evolution on a northwestern Pacific breeding ground: Okinawa, Japan

**DOI:** 10.1098/rsos.241388

**Published:** 2025-02-12

**Authors:** Eleanor M. Marwood, Franca Eichenberger, Nozomi Kobayashi, Haruna Okabe, Sachie Ozawa, Luke Rendell, Ellen C. Garland

**Affiliations:** ^1^ Sea Mammal Research Unit (SMRU), School of Biology, Scottish Oceans Institute, University of St Andrews, St Andrews, Fife KY16 8LB, UK; ^2^ Okinawa Churashima Research Institute, Okinawa Churashima Foundation (OCF), Motobu, Okinawa, Japan; ^3^ Okinawa Churaumi Aquarium, Okinawa Churashima Foundation, Motobu, Okinawa, Japan; ^4^ Centre for Social Learning and Cognitive Evolution, School of Biology, University of St Andrews, St Andrews, Fife KY16 9TH, UK

**Keywords:** song, cultural transmission, cultural evolution, humpback whale, Okinawa, Japan

## Abstract

Male humpback whales (*Megaptera novaeangliae*) sing a slowly evolving, sexually selected song display socially learned from conspecifics. Within an ocean basin, song similarity between breeding populations can reveal the degree of connectivity among them. In the northwestern Pacific Ocean, there is a paucity of information on song dynamics and linkages across the ocean basin. Here, we quantified fine-scale song evolution in whales near Okinawa, Japan, using similarity indices (Levenshtein distance and Dice’s similarity) and song complexity measures to investigate three consecutive years (2011–2013) of song dynamics on this breeding ground. Matched song themes revealed minimal evolution between 2011 and 2012, while the 2013 song was more distinct, as singers sang both new and evolved versions of themes. This was mirrored by the song complexity scores, which decreased and then increased over time. Qualitative comparisons of Okinawa song themes to other published North Pacific breeding ground songs revealed many themes were shared across the North Pacific, contributing to the growing body of evidence of a single panmictic song lineage across the North Pacific Ocean basin, in contrast to the South Pacific. Understanding geographically differing song dynamics is essential to revealing the underlying drivers of this ocean basin-wide non-human culture.

## 1. Introduction

Cultural transmission, the social learning of information or behaviours from conspecifics within a group, leading to group-specific behaviour [1–4], has been characterized in multiple taxa across the tree of life, including humans [5], primates [6], birds [7,8] and cetaceans [9]. As direct visual or acoustic contact may be required for some behavioural traits to pass between individuals, increasing our knowledge of the patterns of cultural transmission can aid our biological understanding of connectivity between populations of a species. The spread of behaviours through social learning between individuals and subsequently throughout a population can occur horizontally and vertically, within and between generations, or obliquely between unrelated conspecifics of different generations [2,10]. Such documented behaviours include innovative feeding techniques, such as the use of stones to crack nuts by chimpanzees (*Pan troglodytes*) in West Africa [6], the use of marine sponges by bottlenose dolphins (*Tursiops* sp.) to protect their rostrum while foraging for food on the seafloor in Shark Bay, Australia [11,12], or the use of ‘lobtail feeding’ by a population of humpback whales (*Megaptera novaeangliae*) in the Gulf of Maine, USA [13].

Recording and characterizing the flow of socially learned behaviours throughout populations can provide an opportunity to rapidly census the level of connectivity among them [14,15]. Analyses of such behaviours may therefore allow inferences of population structure more quickly and cheaply than using molecular approaches, which require the collection of tissue samples and costly laboratory processing, or by photo-identification techniques that require expensive extended periods on research vessels [16]. This is particularly true for species that are difficult to directly observe in the field due to their habitat, life history traits or migration patterns, such as cetaceans.

In the ocean, male humpback whales sing a complex, stereotyped, repetitive, extended song display. The exact function of song in sexual signalling of humpback whales is debated, although the prevailing hypotheses are that song functions in advertising male quality to prospective female mates, plays a role in male–male interactions at wintering grounds, potentially to establish hierarchies among singers, and/or aids individual or population recognition [17–21]. Song could also be multi-functional, serving several purposes in humpback whale reproduction [18]. Most males within a population sing the same version of the song (content and arrangement) at any one time [22,23]. Songs within a population gradually change within and between breeding seasons, as singers alter the duration, structure or frequency content of their sequence of vocalizations, leading to modifications that accumulate over time, termed ‘song evolution’. Due to the strong cultural conformity within the population, males appear to match song changes at a population level.

The structure of humpback whale song itself is hierarchical [24]: the lowest level is the ‘unit’, which is the shortest sound to our ears, while a few units are combined to create a ‘phrase’ and can be unambiguously delineated by identifying the largest gap between units [25]. Phrases are repeated to make a ‘theme’, and multiple themes compose a ‘song’, which is usually 5−30 min in length. Repetition of the same song during a song session can last many hours, and each repetition is referred to as a song cycle. Songs can be categorized into song types depending on their phrase and theme content [26].

Singing generally occurs on low-latitude breeding grounds, to which individuals migrate in the winter from their summer feeding grounds at higher latitudes [27–29]. However, males also sing on migration and, to a lesser extent, on feeding grounds [30–32]. While acoustic isolation of singers at their breeding grounds can allow their songs to diverge, song learning and convergence of song can occur within or between breeding seasons by individuals moving between populations or on shared migration routes or feeding grounds [21,26,28,31–38]. However, humpback whales show strong maternally directed site fidelity to both their breeding and feeding grounds [29].

In addition to song evolutions, cultural ‘revolutions’ have been documented in the Southern Hemisphere, most notably in populations from the South Pacific [26,33,39,40]. Song revolutions involve the rapid and complete replacement of the song type (i.e. all themes) with a novel song type introduced by a neighbouring population [26,39]. Song revolutions occur much faster than the gradual accumulation of small modifications that characterize song evolutions [22,26,39].

In comparison to the South Pacific, North Pacific populations show a relatively high degree of song sharing among singers across breeding grounds, within and between breeding seasons, indicative of a high level of mixing among them [37,41–44]. The most recent analysis, conducted by Darling *et al*. [36], used song recordings from breeding grounds off the Philippines, Ogasawara (Japan), Hawaii and Mexico between 2011 and 2013. The results were consistent with earlier studies [41,42,44], suggesting that the same song lineage is shared and evolves among singers across North Pacific breeding populations, with no evidence for the occurrence of rapid cultural revolutions [36]. Song analyses provide an indication of the level of connectivity between social clusters and have the potential to help inform management of humpback whales [16,36]. Despite some populations recovering from whaling, humpback whales are still subject to other anthropogenic threats, including disturbance of their behaviours from whale-watching tourism and vessel noise, vessel strikes, pollution and entanglement with fishing gear. There is a need for fine-scale analyses of North Pacific humpback whale song, both to aid our understanding of the above stressors and to investigate the level of connectivity between understudied endangered breeding subpopulations (such as the Northwestern Pacific region, including Okinawa) and others across the wider region [36,37,45–48].

Here, we quantified song evolution and song dynamics over 3 consecutive years (2011−2013) in an endangered and relatively understudied subpopulation of humpback whales in Okinawa, Japan, Northwestern Pacific [45–47]. First, we assessed the fine-scale evolutionary change of song within Okinawa across years using a combination of song complexity and similarity analyses. Second, to investigate the spatial dynamics of song across the wider North Pacific, we qualitatively matched song themes from Okinawan humpback whales to humpback whale song themes from Darling *et al*. [36], recorded on breeding grounds off Japan, the Philippines, Hawaii and Mexico, for each year of the study period. We expected, given previous literature, that songs from Okinawan waters would reflect those heard in the wider North Pacific.

## 2. Material and methods

### 2.1. Acoustic recordings

**Figure 1.**
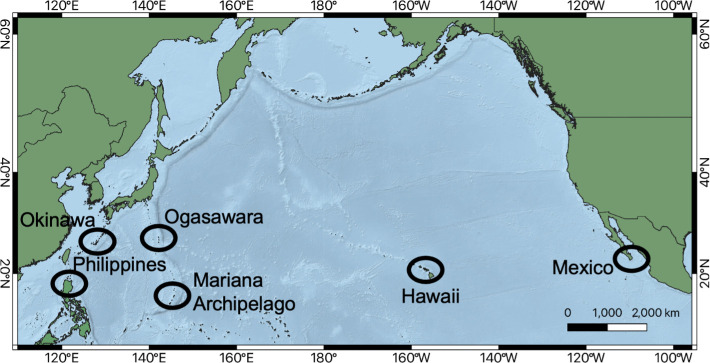
Map showing Okinawa, Japan and the other major breeding grounds across the North Pacific.

Humpback whale songs were recorded using dipping hydrophones and recorders deployed from small vessels. Recordings from 2011 and 2012 were made using a KORG MR-2 recorder and OKI Whale-Phone hydrophone (T1020, Oki Electric Co. Ltd., Japan; frequency range: 10 Hz–150 kHz and sensitivity: −180 dB re 1V/µPa), while recordings in 2013 were made with a SONY PCM Recorder D-10 and Aquasound AQH-020 hydrophone (QH-020, AquaSound Inc., Japan; frequency range: 10 Hz–20 kHz, sensitivity: −193 dB re 1V/µPa; all WAV format, 16-bit, sampling rate 44.1 kHz). Song analyses were limited to 10 Hz–20 kHz across the 3 years. All recordings were made between January and March from 2011 to 2013, during the boreal winter breeding season [46] (table 1). Recordings were made at two sites: the Motobu area and the Kerama Islands, Okinawa, which are approximately 50 km apart from each other, both southwest of Japan in the Northwestern Pacific ([47]; figure 1 and electronic supplementary material, figure S1; for finer scale recording locations and extended methods see electronic supplementary material, S1). The Okinawa subpopulation (including Motobu and the Kerama Islands) is part of the wider breeding population that also includes Ogasawara and the Philippines [49].

**Table 1.** Song recordings from Okinawa, Japan from 2011, 2012 and 2013. The sequence of themes sung (no repetition of a theme but allowing repetition of phrase types if consecutive) is noted per singer (ID), along with the average song complexity score. Theme descriptions are provided in electronic supplementary material, table S1.

year	ID	location	date	length (mm:ss)	#song cycles	theme sequences sung	average song complexity
2011	1	Kerama	16 February	10:34	2	1a, 2, 3a, 4a, 5a, 5b; 1a, 3a, 4a, 5a, 2	−1.1223
	2	Kerama	01 March	11:06	2	3a, 4a, 5c, 5b, 2, 1a; 2, 6, 3a, 4a, 5c, 5b, 1a	1.0589
	3	Kerama	18 March	17:15	2	4a, 5a, 5c, 5a, 5c, 5a, 5c, 5a, 5c, 5a, 5c, 5b, 5c, 5b, 6, 1a, 3 , 2; 5a, 5c, 5b, 6, 3a, 1a	0.3079
	4	Motobu	09 February	12:25	2	4a, 3b, 1b, 2, 3a; 1b, 3a, 4a, 5a, 5c, 5a, 5c, 5a, 5c, 5a, 5c, 5a, 5c, 5a, 5d	−0.1308
	5	Motobu	24 February	16:30	1	1a, 4b, 4a, 3c, 3a	−0.7773
	6	Motobu	05 March	10:15	2	1a, 3a, 4a, 5a, 5c, 5a, 5c, 5a, 5c, 5a, 5c, 5b, 6; 3a, 3c, 4a, 5a, 5c, 5b, 6	0.7445
2012	8	Kerama	29 January	13:09	2	1e, 7a; 1e, 7a, 4a, 5a, 5c, 5a, 5c, 5be	−1.0129
	9	Kerama	15 February	12:05	2	7a, 1e; 7a, 1e, 4a, 5c, 5a, 5c, 5a, 5c, 5a, 5c, 5a, 5c, 6e	−1.4898
	10	Kerama	13 March	13:29	2	1e, 6e, 7a; 1e, 6e, 7a, 5c	−1.9019
	11	Kerama	25 March	13:10	2	6e, 7a, 1e; 7a, 1e, 6e	−2.6698
	12^a^	Motobu	06 March	10:18	<1	7a, 7b, 1e	n/a
	15	Motobu	27 March	12:51	2	6e, 7a, 7b, 1e; 6e, 7a, 4a, 5ce, 5ae, 5ce, 5ae, 5ce, 5ae, 5ce, 5ae, 5ce, 5ae, 5ce, 5ae, 5ce, 5ae, 5ce, 5ae, 5ce, 5ae, 5ce, 5ae, 5be	−0.5086
2013	16	Kerama	06 March	10:19	1	1ee, 6e, 8a, 8b, 8c, 9a, 9b	3.6689
	17	Kerama	16 March	15:05	2	9a, 1ee, 6e, 8a, 8b, 8c; 9a, 1ee, 6e, 8a, 8c	0.6905
	19	Kerama	26 March	10:26	2	8a, 8b, 8c, 9a, 5ae, 5ce, 1ee, 6e; 8 a, 8b, 8c, 9 a, 9b, 5ce, 5ae, 5ce, 1ee, 6e	1.3559
	20	Motobu	08 March	14:10	2	9a, 1ee, 6e, 8a, 8b, 8c; 9a, 5ce, 1ee, 6e, 8a, 8b, 8c	1.6423
	21	Motobu	16 March	10:58	2	9a, 5ce, 5ae, 5ce, 5ae, 5ce, 1ee, 6e, 8a, 8b, 8c; 9a, 1ee, 6e, 8a, 8b, 8c	1.5903
3 years	17 IDs	2 sites	29 January−27 March	3:34:05 (h:m:s)	30 songs	9 themes; 25 phrase types including 6 evolved versions	

^a^ Included in random forest and phrase type assignments. Excluded from song evolution (LSI and DSI) and song complexity analyses due to the lack of a complete song cycle.

### 2.2. Song transcription and unit classification

From the 21 initial recordings, 17 were of high enough quality (with a signal-to-noise ratio (SNR) of song units above 10 dB measured using SNR-NIST Quick in Raven Pro (v. 1.6.4) and units of a single singer clearly distinguishable) to be included in further analysis. Also, following previous studies, recordings from the same location had to be separated by at least 24 h to be included in the analysis to minimize the possibility of recording the same individual twice [50]. We endeavoured to include recordings spread across the season to capture the gradual process of song change that occurs, but we only had sufficient data to do this for 2011 and 2012, as 2013 recordings were only from March (table 1).

All songs were visualized as spectrograms (2048 FFT, Hann window, 75% overlap) in Raven Pro (v. 1.6.4). Following previous analyses, songs were transcribed at the unit level by a human classifier (EM) based on the aural and visual qualities of units [26,31,33,40,51]. To ensure this qualitative classification was robust and repeatable across singers and years, a random forest analysis (package *randomForest*; [52]) was run in R (v. 4.4.1). Using 11 acoustic parameters (see electronic supplementary material, S1 [31,40,53–55]) measured from all high-quality units (*n* = 4575) from 17 recordings, the random forest (mtry = 3, ntrees = 10 000) had an out-of-bag error rate (OOB) of 16.35%, indicating the qualitative naming and classification of song units was robust and repeatable.

### 2.3. Assigning phrases and themes

Phrases were transcribed from repeated strings of units identified using the largest time interval between units (following [25]), and repeated phrases were labelled as themes (e.g. theme 1), allowing similar sounds in similar positions to be classified as the same theme [25,26,56]. Variations in the units within phrases that occurred consistently (e.g. the replacement of a ‘groan’ with a ‘moan’ or the absence/presence of a unit) were labelled as different ‘phrase types’ (e.g. theme 1 could have two phrase types: 1a and 1b). Instances of song evolution where phrases had changed consistently by one or two units between years were labelled with an ‘e’ (e.g. theme 1e). Two full song cycles were transcribed, where possible, for each singer. In line with previous humpback whale song analyses, song cycles were defined as a sequence of themes with no repetition [22]. When singing one variable theme (theme 5), individuals routinely alternated between different phrase types (5a, 5b, 5c, 5d and the later versions of 5ae, 5be, 5ce, 5de) multiple times before moving on to the next theme. A new song cycle was not designated until sequences of alternating phrase types from this theme had ended (table 1).

To ensure that phrase types were assigned correctly and consistently, strings of units within phrases were compared using the Levenshtein Distance Similarity Index (LSI), following previous studies [31,40,51,54,55,57,58]. Briefly, LSI calculates the minimum number of insertions, substitutions or deletions required to turn one sequence into another, standardized by the length of the longest string (for further details see electronic supplementary material, S1) [51,54,55,59,51,54,55,59]. This produces a matrix of similarity (between 0, no similarity, and 1, identical strings). The LSI analysis was conducted in R using custom-written code (package *leven*, available at http://github.com/ellengarland/leven) to check the assignment of phrase types (*n* = 1687 phrases, from 17 singers). The similarity matrix was then hierarchically clustered using average-linkage (UPGMA) clustering and displayed as a dendrogram to ensure consistent classification. Finally, using the ‘cophenetic’ function from the *stats* package (in R), we calculated the cophenetic correlation coefficient (CCC) to check the degree to which the dendrogram represented the structure of the data. This resulted in a CCC of 0.93, where CCC > 0.8 is considered ‘good’ [54,60], indicating our phrase-type assignments were robust and were a very good representation of the connections within the data.

Once phrase types were confirmed, we calculated median strings (the most typical or representative string in a set, here a sequence of units of the same phrase type) for each phrase type per singer and per year. These median strings, or ‘set medians’ (for set medians, see electronic supplementary material, table S1), were then available for the qualitative comparison of song themes across the North Pacific (see below). The sequence of phrase types (themes) within each song per singer was also noted (with phrase repetitions removed; table 1). Theme sequences per year were visualized in transition diagrams to display the variability in these transitions as the song evolved through time.

### 2.4. Quantifying Okinawa song evolution

#### 2.4.1. Song similarity analyses

To investigate the level of similarity among the songs of different singers, two similarity analyses were conducted: the LSI and Dice’s Similarity Index (DSI). The LSI was run on the median theme sequence (*n* = 16, calculated in the *leven* package) per singer. The theme matrix was hierarchically clustered using average-linkage (UPGMA) clustering and bootstrapped (package *pvclust* [61]) 1000 times (with multiscale bootstrap re-sampling (AU), significant if *p* > 95%, and normal bootstrap probability (BP), significant if *p* > 70%) to ensure the structure produced was stable [51]. To further check the structure reflected the connections in the data, CCCs were also calculated using the method described above.

Dice’s Similarity Index (DSI) was calculated using full strings of phrase types per singer (songs were not split into song cycles with a median string; *n* = 16) using the custom-written package *dice_si* (available at http://github.com/ellengarland/dice_si) in R. Here, DSI measured the extent of theme sharing among singers irrespective of their order (for further details see electronic supplementary material S1). The DSI matrix was clustered (with average linkage) as above and bootstrapped 1000 times to ensure its structure was robust and stable, and CCC was also calculated.

#### 2.4.2. Song complexity analyses

To further investigate song evolution through time, we conducted a song complexity analysis. Although our sample size was limited, we decided to pursue this analysis because it offers valuable insights into population-level humpback whale song dynamics in the understudied western North Pacific. We calculated humpback whale song complexity scores per song for each year following [50,62], which was adapted from scores calculated for song complexity in zebra finch (*Taeniopygia guttata*) [63,64]. While the complexity measures were first constructed for zebra finch song which is much shorter and has less unit repetition than humpback song, our previous analyses have confirmed that the measures appear robust to increased duration and increased unit repetition [50,62]. Briefly, we measured four song variables: number of units per song, number of unit types per song, duration (in seconds) of each song and number of themes per song (from *n* = 1650 phrases, *n* = 30 songs, *n* = 16 singers). All statistical analyses were conducted in R (v. 4.2.1). Variables were checked for normality using a Shapiro–Wilk normality test; three variables (number of units, number of unit types and duration) were log-transformed to improve normality, while the left-skewed variable number of themes was square transformed to improve normality. The four variables included in all further analyses were the log number of units, the log number of unit types, the log duration and squared themes. Hereafter, we refer to these variables as the number of units, number of unit types, duration and themes.

The relationships among the four variables were checked using a Pearson’s correlation test. All variables were positively correlated with varying degrees. The number of themes was positively correlated with the number of unit types (*r* = 0.631, *p* < 0.001), the number of units (*r* = 0.753, *p* < 0.001) and the duration of each song (*r* = 0.419, *p* = 0.021). The duration of each song was positively correlated with the number of units (*r* = 0.796, *p* < 0.001) and the number of unit types (*r* = 0.383, *p* = 0.037). Finally, the number of unit types was positively correlated with the number of units (*r* = 0.572, *p* = 0.001).

Next, a principal components analysis (PCA) was run, following [50,62–64], using the *princomp* function and the first principal component extracted for each song as the ‘song complexity score’. To reduce overrepresentation of singers with more data, we averaged the song complexity scores per individual. This produced an average song complexity score per singer (table 1). Homogeneity of variance among years was checked for completeness. Finally, to assess whether the complexity scores were significantly different across the 3 years within this single song lineage, a linear regression model was run.

### 2.5. Qualitative comparison of Okinawa songs with other North Pacific songs

To compare the song of Okinawa humpback whales to that of the wider North Pacific from 2011 to 2013, the median strings of themes from each year in the current study were visually (spectrograms) and aurally (audio files) compared with humpback whale song from Darling *et al*. [36], which included the breeding grounds off Japan, the Philippines, Hawaii and Mexico.^1^ The Japanese study site in Darling *et al*. [36] was Ogasawara, 1430 km east of Okinawa; there is a relatively high degree of interchange of individuals between these islands and with the Philippines, and the Mariana archipelago with individuals at these four breeding grounds regarded as the same breeding population (figure 1) [46–48]. All qualitatively matched themes (not distinguishing the finer-scale differences in phrases or evolutions of those phrases within themes) were confirmed by two authors (E.G. and E.M.).

## 3. Results

### 3.1. Okinawa song structure and theme transitions

**Figure 2.**
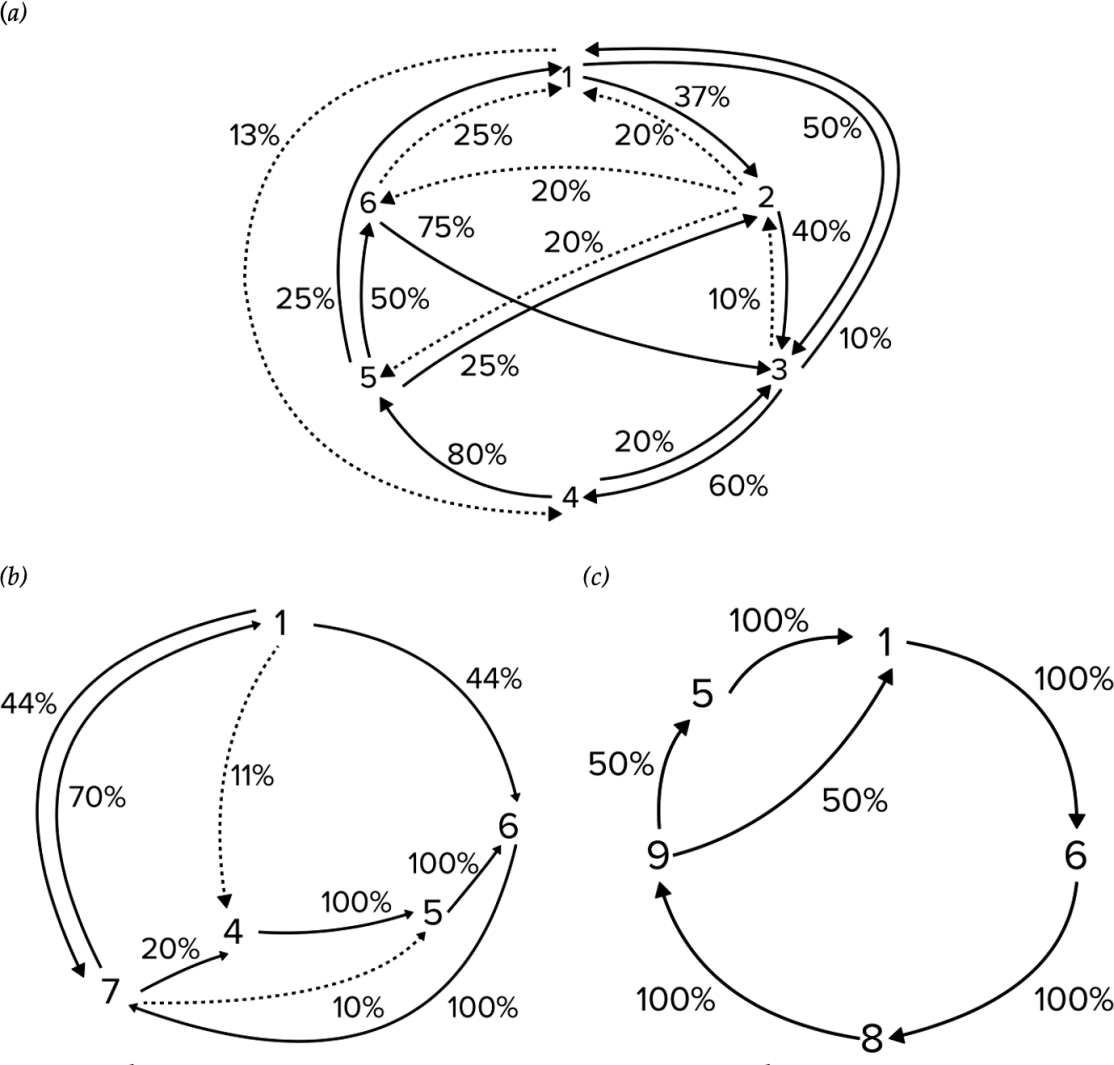
Diagrams showing transitions within Okinawan songs in different years: (*a*) 2011, (*b*) 2012 and (*c*) 2013. The sequencing of themes within the songs became progressively more stereotyped across the study period. Transitions only observed once are represented with a dotted line. Percentages show the proportion of the time that each transition occurred from a given theme.

From the 17 recordings (referred from here on as individual singers) spread over 3 years (2011−2013), a total of 7507 units were transcribed, grouped into 1687 phrases that were sung in over 30 song cycles (table 1). The 3 years of song data contained a total of nine themes (described in detail in the electronic supplementary material and table S1). We acknowledge that this represents a snapshot of song at each point in time with a small number of singers. However, all singers appeared to sing different versions of the same song, as themes recorded in multiple years were observed in each of the breeding seasons analysed (figure 2), suggesting the sample was broadly representative of song at this location.

Over the 3 years, the structure of the song appeared the least stable in 2011 (figure 2*a*). During this breeding season, although many themes were shared, the order in which they were sung varied within and among singers, such that no two song cycles in our small sample showed the same sequence of themes. The most common transitions occurred between themes 3 and 4, and themes 4 and 5. However, the song transitions became less stable outside of these themes, as most other combinations were observed. Although generally more stable than the 2011 song, parts of the 2012 song were more variable in their transition structure than others (figure 2*b*). Just under half of the transitions from theme 1 were to theme 6 or theme 7, although the positions of themes 1 and 7 alternated in different song cycles. The song was the most stable in terms of its transition between themes 6 and 7, after which, similar to 2011, the structure of the song then became more variable in theme 5. Although theme 5 was sung by all but one singer in 2012, this theme was absent in half of the song cycles analysed. Songs recorded in 2013 showed the highest stability in their theme sequence structure (figure 2*c*). Song cycles in 2013 differed only in whether theme 5 was sung or not. Otherwise, transitions between other themes were consistent, stable and predictable across all 2013 singers.

### 3.2. Okinawa song evolution

#### 3.2.1. Song similarity analyses

**Figure 3.**
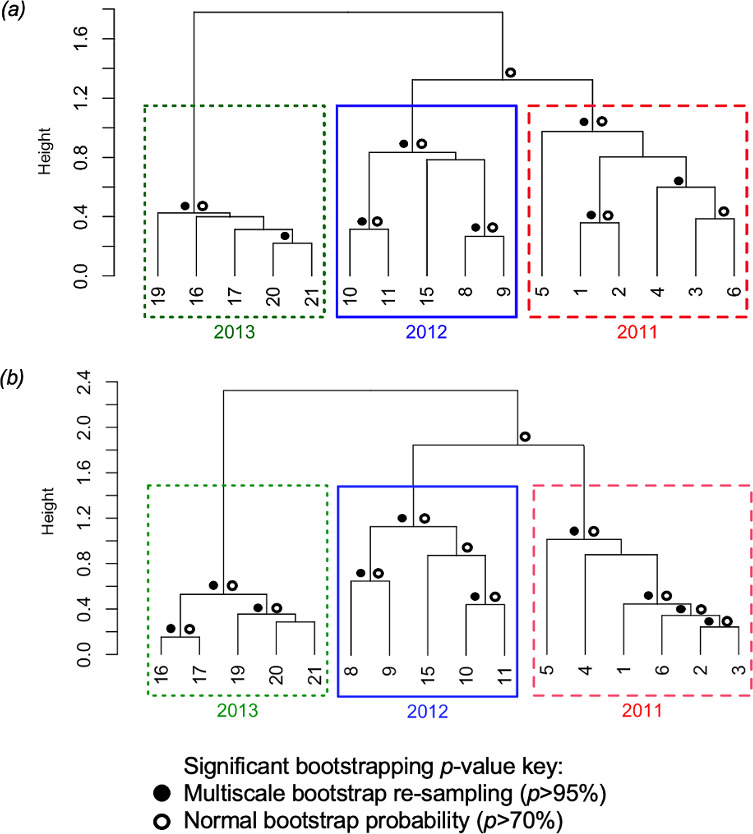
Dendrograms of bootstrapped (1000) average-linkage hierarchically clustered Okinawa songs recorded from 2011 to 2013 using (*a*) LSI using set median theme sequence (CCC = 0.961) and (*b*) DSI using theme presence (CCC = 0.916, table 1). Boxes highlight singers clustering within years 2011 (dashed red), 2012 (solid blue) and 2013 (dotted green).

Two major song clusters were resolved in the LSI dendrogram (average linkage; figure 3*a*). One branch included two groupings with singers from 2011 and 2012, respectively. The second major cluster in the dendrogram included all individuals from 2013. Similar to LSI, hierarchically clustering the DSI matrix, which considered phrase type (theme) sharing regardless of sequence, showed the same pattern (figure 3*b*).

Overall, the results of hierarchical clustering of both the LSI and DSI matrices suggested that there was higher similarity in the songs of Okinawa humpback whales within years than between them. This provides evidence that the song evolved between breeding seasons and suggests the 2011 and 2012 songs were more similar to each other than to the 2013 song, which contained changed versions of many themes. The song in 2013 was clearly the most distinct of the three breeding seasons analysed, both in terms of which themes were sung and in terms of theme sequence (figure 3).

#### 3.2.2. Song complexity analyses

**Figure 4.**
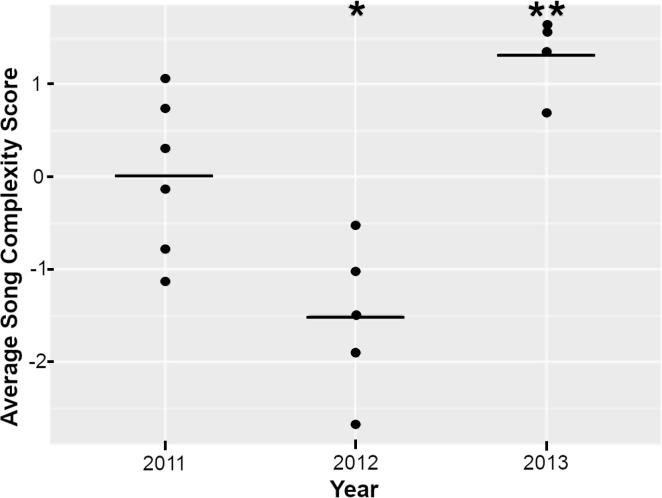
Okinawa song complexity through time (2011−2013). Complexity scores were calculated from four song measures: # themes, # unit types, # units and duration of each song. Each dot represents the average song complexity score per singer per year (table 1), and the line represents the model estimated means per year. The linear regression indicated song complexity significantly changed through time (Adj. *R*^2^ = 0.661, F-statistic = 15.63, d.f. = 2 and 13, *p* < 0.001), with a significant decrease in complexity in 2012 (*p < 0.05) and a significant increase in complexity in 2013 (**p < 0.01).

The PCA of the four song variables resulted in a single principal component (PC1) that explained 69.9% of the variance. The component loadings on PC1 were 0.564 for the number of units per song, 0.457 for the number of unit types per song, 0.466 for the duration and 0.506 for the number of themes present per song. The scores for PC1 for each song were extracted and averaged per singer to represent the complexity score for that song (table 1). Homogeneity of variance of complexity scores among years was then checked (Bartlett test, K-squared = 0.425, d.f. = 2, *p* = 0.809). Song complexity changed through time, first by decreasing and then increasing within the single song lineage (figure 4). Overall, song complexity scores were significantly different among the 3 years (Adj. *R*^2^ = 0.661, F-statistic = 15.63, d.f. = 2 and 13, *p* < 0.001). Song complexity was significantly lower in 2012 compared with 2011 (*t*-value = −2.700, *p* = 0.018), followed by a significant increase in complexity in 2013 (*t*-value = 3.134, *p* = 0.008).

### 3.3. Qualitative comparison of Okinawa songs with other North Pacific songs

**Figure 5.**
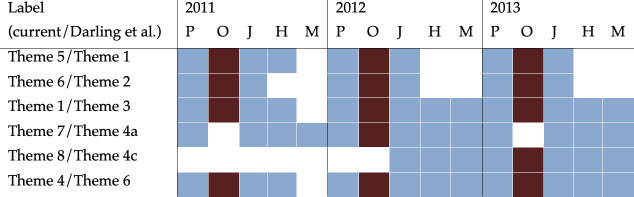
The presence of shared themes in 2011, 2012 and 2013 in Okinawa (*O*) (brown) and in Darling *et al*. [36] (blue), in the Philippines (*P*), Ogasawara (Japan) (*J*), Hawaii (*H*) and Mexico (*M*). All proposed matches were aurally and visually confirmed following comparison of recordings between Okinawa and those in Darling *et al*. [36], provided by first author J. Darling.

Through visual and aural comparisons of themes (see electronic supplementary material, table S1 for a representative set of median unit sequences used in comparisons) recorded each year at Okinawa, and of themes published in Darling *et al*. [36], a minimum of six themes were suggested to match across both studies (figure 5; for spectrograms of matched themes see electronic supplementary material, figure S2). The presence of shared themes varied among breeding grounds and years, with fewer shared themes between Okinawa and other North Pacific breeding grounds in 2011 compared with 2012 and 2013 (figure 5). For instance, Theme 8 (this study)/Theme 4c [36], which was absent across the region in 2011, first appeared in Mexico, Hawaii and Ogasawara in 2012, but only reached the westernmost breeding grounds of Okinawa and the Philippines in 2013 (figure 5). All six shared themes identified in Okinawa were observed in Ogasawara and the Philippines by Darling *et al*. [36] at least once between 2011 and 2013 (figure 5). Out of four shared themes present in Okinawa, the Philippines and Ogasawara from 2011 to 2013, two were also present in Hawaii in all years (T1/T3 and T4/T6) and arrived in Mexico in 2012, while one (T6/T2) was not recorded by [36] in Mexico, and the other (T5/T1) only occurred in Hawaii in 2011. In Hawaii, five of the six shared themes we identified in Okinawa were also observed by Darling *et al*. [36], while only four of the six shared themes were observed in Mexico, which shared the fewest themes with the songs from Okinawa.

## 4. Discussion

Here, using a combination of song complexity and similarity analyses, we have shown fine-scale evolutionary song change and dynamics over three consecutive years (2011−2013) of humpback whale song from Okinawa, Japan. This provides a baseline for future song studies in this endangered and relatively understudied Northwestern Pacific subpopulation [45,47]. In addition, qualitative comparisons of Okinawa song themes to song themes from other North Pacific breeding grounds (recorded from 2011 to 2013, published in Darling *et al*. [36]) showed that large portions of the Okinawa song (six out of nine themes) were observed at other North Pacific breeding grounds within the same time period, including Ogasawara (Japan), the Philippines, Hawaii and Mexico. This work contributes to the growing body of evidence of a single song lineage across the North Pacific Ocean basin, suggesting consistent and persistent acoustic contact between all studied North Pacific populations, in stark contrast to the Southern Hemisphere [26].

Okinawa song progressively evolved from 2011 to 2013. It was clear that themes were added, evolved and deleted as time progressed (table 1). Furthermore, the structure of the song became more predictable and stable as time progressed (figure 2). The observed patterns were common among individuals within years; for instance, all 2011 singers appeared to vary in the sequence of the themes they sang, while all 2013 singers showed more similarity in their song structure (table 1). Both LSI and DSI quantitative analyses revealed singers clustered within the years they were recorded, and that 2011 and 2012 singers were more similar to each other than to 2013 singers (figure 3). Clustering suggested the Okinawa song in 2013 was most distinct, likely due to the presence of themes 8 and 9 (figure 3, table 1).

Our complexity analysis revealed a significant decrease followed by a significant increase in complexity as the song changed over the 3 years. Results of this analysis should be interpreted with some caution due to the small sample size but are still valuable to explore in light of our understanding of complexity measures in song. Previous studies from the Southern Hemisphere have shown that song complexity scores increased between revolution events and decreased during them [50,62,65]. In the North Pacific, song revolutions have thus far never been documented; therefore, we did not expect to see similar complexity dynamics. We did, however, see complexity change across the 3 years (figure 4) in a manner similar to the evolution/revolution dynamics of Southern Hemisphere song, but herein within the same single song lineage. However, the data presented herein are a snapshot in time and are limited in sample size; future studies with larger sample sizes (both with more song cycles per singer and years investigated) are required to understand the dynamics of a single song lineage and how its progressive evolution translates into complexity. We hypothesize that Northern Hemisphere song complexity will fluctuate sporadically around a median complexity level. Peaks in complexity may represent an upper limit of song learning, as suggested in [50,62]. We hypothesize that decreases in complexity may correspond to substantial changes in song material demonstrated as song revolutions in the Southern Hemisphere, or alternatively, may simply reflect that the song is ‘simpler’ (as song revolutions have been shown to be significantly less complex than the preceding song [50,62]). Understanding geographically differing song dynamics is essential to revealing the underlying drivers of this ocean basin-wide non-human culture and clarifying how such drivers integrate with its suggested function as a sexually selected display.

The presence of shared themes among humpback whales at Okinawa and other North Pacific breeding grounds is consistent with previous song analyses, suggesting that acoustic contact likely occurs frequently enough to prevent songs from diverging, thus maintaining a single evolving song lineage across the North Pacific [36,37,66,67]. Okinawa humpback whales shared the most themes with singers from Ogasawara and the Philippines, with fewer shared themes with songs from Hawaii and Mexico (figure 5). This would be expected as individuals at Okinawa, Ogasawara and the Philippines are regarded as the same breeding population [49]. Fewer shared themes were identified between Okinawa and other North Pacific breeding grounds in 2011 compared with 2012 and 2013 (figure 5). These results indicate that increased acoustic contact between singers from Okinawa and other breeding grounds occurred after the 2011 winter, increasing song learning opportunities among them. The driver for the suggested decreased mixing in 2011 is unknown, but future studies should integrate acoustic similarity analyses with satellite tracking and other methods to identify similar patterns.

One of the main factors, however, thought to be contributing to the homogenization of the entire North Pacific song, is population overlap and contact on the feeding grounds [68,69]. Humpback whales from Okinawa, Ogasawara and the Philippines have been identified in feeding grounds off Russia, the Aleutian Islands and in the Bering Strait, where individuals from Hawaii and Mexico have also been observed [28,70]. Although song is typically associated with breeding grounds, singing has been documented at feeding grounds in the North Pacific, the western and eastern North Atlantic, and on Antarctic feeding grounds in the Southern Ocean, where evidence suggests that song sharing has occurred between South Pacific populations [32,35,58,71–74]. The plausibility of frequent acoustic contact in facilitating the homogenization of North Pacific song has been theoretically demonstrated by modelling studies of song transmission across the ocean basin, where continental land masses funnel populations into a relatively small area on high-latitude feeding grounds [67]. Therefore, the mixing of singers at their feeding grounds and on migration likely allows song sharing among them, maintaining a single song lineage across the North Pacific. Future studies that compare songs recorded from multiple feeding grounds with multiple breeding grounds are required to pinpoint the directionality and speed of song sharing across this ocean basin.

The apparently high levels of acoustic exchange inferred from song analyses contrast with results from mtDNA haplotype analyses showing strong genetic differentiation between breeding grounds [29]. However, this discrepancy could be due to differences between the sexes, as mtDNA passes down female lineages, but song lineages are male-mediated. Indeed, the mtDNA study noted that there was evidence of a male-biased gene flow across the North Pacific, which could explain song sharing, as song is a very sensitive indicator of contact between populations, being much easier to exchange than genes [29]. Finally, understanding the level and extent of mixing has potential management implications; the current study adds to the growing body of evidence indicating that considering North Pacific breeding populations separately may not be appropriate [36]. Future studies assessing the connectivity among breeding grounds using song, genetic, satellite tracking and photo-ID data are required, highlighting the difficulty in managing such a wide-ranging and highly migratory species.

## 5. Conclusion

In conclusion, using a combination of song complexity and similarity analyses, this study provided a robust quantitative analysis of fine-scale evolutionary song change and dynamics over three consecutive years (2011−2013) of humpback whale song from Okinawa, Japan. Matched song themes revealed nominal change between 2011 and 2012, while the 2013 song had substantially evolved. Song complexity scores that both increased and decreased through time appeared to mirror the revolutionary dynamics of complexity in the South Pacific. This study contributes to the growing body of evidence of a single, evolving song lineage across the North Pacific Ocean basin, likely due to the relatively high degree of acoustic connectivity among them at their feeding grounds and possibly on their migration routes. This is in contrast to the Southern Hemisphere, which experiences dynamic (revolutionary) song changes. Understanding geographically differing song dynamics is essential to revealing the underlying drivers of this ocean basin-wide non-human culture and clarifying how such drivers integrate with its suggested function as a sexually selected display.

## Data Availability

The datasets supporting this article have been uploaded as part of the supplementary material [75].

## Footnotes

^1^
Audio files of all potential matched themes in [36] were supplied by Jim Darling, January 2024, to allow both aural and visual confirmation of themes with Okinawa song.
